# Effect of Citric Acid Hard Anodizing on the Mechanical Properties and Corrosion Resistance of Different Aluminum Alloys

**DOI:** 10.3390/ma17174285

**Published:** 2024-08-29

**Authors:** José Cabral-Miramontes, Facundo Almeraya-Calderón, Ce Tochtli Méndez-Ramírez, Juan Pablo Flores-De los Rios, Erick Maldonado-Bandala, Miguel Ángel Baltazar-Zamora, Demetrio Nieves-Mendoza, María Lara-Banda, Gabriela Pedraza-Basulto, Citlalli Gaona-Tiburcio

**Affiliations:** 1 Universidad Autónoma de Nuevo León, FIME, Centro de Investigación e Innovación en Ingeniería Aeronáutica (CIIIA), San Nicolás de los Garza 66455, Mexico; jose.cabralmr@uanl.edu.mx (J.C.-M.); facundo.almerayacld@uanl.edu.mx (F.A.-C.); maria.laraba@uanl.edu.mx (M.L.-B.); 2 Facultad de Ingeniería Civil, Universidad Veracruzana, Xalapa 91000, Mexico; erimaldonado@uv.mx (E.M.-B.); mbaltazar@uv.mx (M.Á.B.-Z.); dnieves@uv.mx (D.N.-M.); 3 Tecnológico Nacional de Mexico-Instituto Tecnológico de Chihuahua, Av. Tecnológico 2909, Chihuahua 31130, Mexico; jpdelosrios@uach.mx; 4 Facultad de Ingeniería y Tecnología, Universidad Autónoma del Carmen, Ciudad del Carmen Campeche 24180, Mexico; gpedraza@pampano.unacar.mx

**Keywords:** aerospace alloy, corrosion, anodizing, electrochemistry, aluminum

## Abstract

Hard anodizing is used to improve the anodic films’ mechanical qualities and aluminum alloys’ corrosion resistance. Applications for anodic oxide coatings on aluminum alloys include the space environment. In this work, the aluminum alloys 2024-T3 (Al-Cu), 6061-T6 (Al-Mg-Si), and 7075-T6 (Al-Zn) were prepared by hard anodizing electrochemical treatment using citric and sulfur acid baths at different concentrations. The aim of the work is to observe the effect of citric acid on the microstructure of the substrate, the mechanical properties, the corrosion resistance, and the morphology of the hard anodic layers. Hard anodizing was performed on three different aluminum alloys using three citric–sulfuric acid mixtures for 60 min and using current densities of 3.0 and 4.5 A/dm^2^. Vickers microhardness (HV) measurements and scanning electron microscopy (SEM) were utilized to determine the mechanical characteristics and microstructure of the hard anodizing material, and electrochemical techniques to understand the corrosion kinetics. The result indicates that the aluminum alloy 6061-T6 (Al-Mg-Si) has the maximum hard-coat thickness and hardness. The oxidation of Zn and Mg during the anodizing process found in the 7075-T6 (Al-Zn) alloy promotes oxide formation. Because of the high copper concentration, the oxide layer that forms on the 2024-T6 (Al-Cu) Al alloy has the lowest thickness, hardness, and corrosion resistance. Citric and sulfuric acid solutions can be used to provide hard anodizing in a variety of aluminum alloys that have corrosion resistance and mechanical qualities on par with or better than traditional sulfuric acid anodizing.

## 1. Introduction

Aluminum alloys are essential components for several high-tech industries, the most well-known being aerospace and automotive. Aluminum alloys continue to show exceptional promise for a variety of industrial applications, despite the range of production costs and material science considerations [1]. These alloys are ideal for very specific applications based on their intended purpose; these include low density, excellent relative mechanical qualities, good formability, and superior corrosion resistance [2,3]. Because aluminum alloys require corrosion protection to retain their intended characteristics, the anodizing process is a crucial step in the manufacturing process [4]. An aircraft’s corrosion defense system typically uses a multi-level strategy that includes an organic finish layer, an organic base coat that inhibits corrosion, and an anodized oxide layer that offers good barrier qualities and surface porosity [5]. Numerous varieties of aluminum alloys have been created over time, each having unique properties and uses. Examples of alloys extensively researched and utilized to produce vital parts for cars, aircraft, or other high-performance heavy machinery are alloys 2024, 6061, and 7075. Cooper serves as the main alloying element in alloy 2024; it provides good formability, good strength-to-weight ratio, great fatigue resistance, and good serviceability [6]. These alloys, which are found in fuselage skins, stringers and frames, and the interior structural parts of wings, can have higher specific strengths and lower specific gravities [7,8]. Alloy 6061, which is primarily composed of silicon and magnesium, is easy to work with, easy to weld, and has good resistance to corrosion. It has a primary hardening phase [9] and is used in secondary structural components or non-structural ones like supports or brackets [10]. Of the three aluminum alloys, alloy 7075, with zinc as the main alloying element, has the highest mechanical strength. The metals in question exhibit remarkable resistance to stress corrosion cracking and have a specific strength-to-weight ratio [11]. This alloy is used to make pressure bulkheads, landing gear components, main wing spars, and wing ribs. The 7XXX family is the most widely utilized series in the aviation industry because it has many grades of alloy that are tailored for certain purposes in the manufacturing of aircraft structural sections [12].

Anodizing is an electrochemical process to artificially increase the oxide film on a metal surface, creating an oxide layer that can be several micrometers thick, which gives aluminum alloys resistance to wear and corrosion for a number of industrial applications. The process involves the adsorption of oxygen from the electrolyte solution onto the metal substrate, resulting in the creation of an oxide layer. This artificial oxide film is formed on aluminum when a sufficient voltage or current flows through an electrolyte in which aluminum is the anode and a suitable material is the cathode [13]. Al^3+^ cations and O^2−^ or OH^−^ anions are the mobile species involved in the anodization process of aluminum in aqueous solutions [14,15]. Al^3+^ cations are generated at the aluminum/oxide interface, leading to aluminum oxidation; on the other hand, due to the extraction of H^+^ from H_2_O molecules, O^2−^ and OH^−^ anions are formed at the oxide/solution interface [14]. The potential drop caused by the insulating properties of aluminum oxide at the metal/oxide/electrolyte interface, causes a high electric field [16]. O^2−^ and OH^−^ anions travel to the oxide/metal interface through the film. After that, they react with Al^3+^ cations, producing oxide [15]. Many of the Al^3+^ cations that are available are not consumed at the oxide/metal interface and are directed toward the electrolyte. Al^3+^ cations that have migrated from the oxide/metal interface with the available O^2−^ anions can form additional alumina at the oxide/electrolyte interface [15,17,18]. This causes tiny changes in the metal surface, affecting both its texture and crystalline structure [19,20]. To stop moisture from leaking through their pores and causing corrosion, thick layers must be sealed. This process not only prevents corrosion but also strengthens threaded parts’ resistance to wear, forms insulating layers in electronic components like capacitors, increases paint adherence, and serves as a dielectric substance [21]. In the industrial world, anodizing comes in three primary varieties, each with unique qualities and attributes that fit various uses. Conventional coatings made by a chromic acid bath technique are the basis for chromic acid anodizing (Type I); the aerospace sector frequently uses these coatings due to their excellent paint adherence and corrosion resistance. The most widely utilized method for coloration is sulfuric acid anodizing (Type II), which is composed of coatings from a sulfuric acid bath. Lastly, thicker coatings are produced by hard-coat anodizing (Type III), which involves a bath at low temperatures and high current densities. This produces a surface that is more resistant to wear and abrasion [19,22,23].

Because acidic electrolytes have low acid dissociation constants (pKa), it is known that they form porous oxide layers. The specific adsorption of cations and anions at the oxide–solution interface is known as a surface coordination reaction [24,25]. The hydroxyl group on the surface has an oxygen atom that can act as a donor (Lewis base), which can coordinate with metal ions or protons (Lewis acid); in the surface layer, the underlying aluminum (III) structural ion is a Lewis acid, which can exchange the hydroxyl group for other coordination anions. The specific adsorption at the alumina–water interface of a carboxylic acid can be described as a ligand exchange, where the hydroxyl group is exchanged for a carboxylate group. Based on the ligand exchange model, the adsorption is a simple exchange of the OH^−^ group for an R–COO^−^ group without influence on the surface charge [24,25,26]. These substances are classified as inorganic acids, organic carboxylic acids, and organic cyclic oxocarbonic acids [27]. The two most often utilized acids are chromic acid and sulfuric acid. However, because of their poisonous and corrosive character, these chemical compounds pose serious threats to both human health and the environment. Because sulfuric and chromic acid particles dispersed in the environment can be inhaled without noticing, there is a close connection between pollution and health effects. Sulfuric acid is included in the group of compounds known as carcinogenic, mutagenic, and reprotoxic (CMR) [28]. The Agency for Toxic Substances and Disease Registry (ATSDR) has studied this subject and found some alarming results. According to this research, there was a higher incidence of laryngeal cancer in those who breathed high quantities of sulfuric acid while working. This emphasizes how crucial it is to replace sulfuric acid in industrial operations with safer alternatives [29].

One significant organic substance that is frequently present in nature is citric acid. It was first identified when lemon juice was extracted by adding lime, and it naturally exists in citrus fruits like lemons and pineapples. A variety of industries use citric acid extensively due to its safe composition and its capacity to bind metals, including those associated with food, medicine, chemicals, and metals [30]. Among its many uses, this organic acid is a chelating agent, preservative, oxidizing agent, and pH regulator. It is used in the production of environmentally friendly detergents, metal coatings, chemical cleaning, and water treatment. By adding sulfuric acid, it is possible to recover citric acid from its calcium salt and produce calcium sulfate and free citric acid [31]. Among the organic acids that are used commercially, citric acid is one that is widely used in a variety of industries. Market estimates anticipate a worldwide value of $3.6 billion by 2025, while industry estimations point to a production volume of 2 million tons for 2020 [32].

Studies have been performed on citric acid as a potentially healthier and more ecologically friendly substitute for sulfuric acid. It has demonstrated the capacity to form and provide efficient surface protection for aluminum alloys throughout the anodizing process. It has previously been used to anodize the aluminum alloys 2024 and 6061, showing notable improvements over sulfuric acid in terms of anodic coating thickness and shape. It is therefore a more environmentally friendly choice for anodizing procedures [8,10,29,33].

The addition of citric acid to sulfuric acid electrolytes has been investigated for different purposes, such as to predict the possible compounds formed during the anodizing process, to control the size of the pores generated in the porous layer, to improve the surface morphology of the coatings formed to manufacture capacitors, and above all, to improve the corrosion resistance of the coatings [34,35,36,37,38,39,40]. These works have found that the correlation of aluminum with organic additives, in this case citric acid, is physical, not chemical, since the characteristic bands of carbonyl and hydroxyl in the infrared spectra do not disappear [34]. Referring to the pore size, they have found the formation of large pore size of about 500 nm anodizing with voltages of 200–370 V; this high-voltage anodizing in citric acid causes the formation of a non-uniform anodic oxide with a burnt black oxide film during anodizing [35]. The morphology results show that the high-voltage-anodized oxide film is formed with a high crystallinity inner layer and a low crystallinity outer layer. However, the crystallinity of the film formed in a mixed solution of boric acid plus citric acid is higher than that of the film formed in boric acid solution only, leading to an increase in the field strength of the film and its special capacitance. In this work, an anodizing voltage of 530 V, a boric acid solution, and a solution composed of boric acid plus citric acid [36] are used. Regarding corrosion resistance, all the works agree that the corrosion resistance of anodized aluminum is increased with mixtures of sulfuric acid and citric acid additions [37,38,39,40]. However, in all these works, citric acid is an additive to the main solution formed by sulfuric acid.

The research objective is to see how citric acid affects the microstructure of the three distinct aluminum alloys 2024-T3, 6061-T6, and 7075-T6, the mechanical properties, the corrosion resistance, and the hard anodic layers’ morphology and to determine and analyze the qualities of the protective coating formed by the anodizing bath solutions of citric acid and mixtures of citric and sulfuric acid using two different current densities. Since these alloys are widely used in the aeronautical industry due to their properties, these three aluminum alloys were chosen for the study. Since they have intermetallic phases rich in copper (2024-T3) with Al-Cu-Mg-Si intermetallics, aluminum alloy 6061-T6 presents phases composed of Fe-Si-Mn, and aluminum alloy 7075-T6 with fine phases composed mainly of Al-Mg-Zn-Cu. Each alloy produces different types of precipitates, which significantly affect the properties of the anodic coatings formed with citric acid and mixtures of citric and sulfuric acid. Electrochemical characterization of these alloys could find potential in aeronautical applications such as fuselage, turbine blades, aircraft landing gear, and structural components. The alloys of aircraft are susceptible to localized or general corrosion when they are exposed to different atmospheres such as industrial (acid rain, H_2_SO_4_) and marine (NaCl).

## 2. Materials and Methods

### 2.1. Materials

The materials selected were aeronautical aluminum alloys 2024-T3, 6061-T6, and 7075-T6 used in the received condition. A 50 ± 2.5 mm diameter bar, in the form of 7 ± 0.25 mm thick discs, was cut to be used as samples before anodizing. X-ray fluorescence was used to determine each aluminum alloy’s chemical composition (Olympus DELTA XRF., Richmond, TX, USA).

The sample preparation was realized employing metallographic methods in accordance with ASTM E3 and E407 [41,42]. The polishing was carried out with various SiC abrasive paper grades: 180, 240, 360, 400 and 600. Before the anodizing procedure, they were ultrasonically cleaned in ethanol for ten minutes after polishing and air-dried.

### 2.2. Hard Anodizing Process

The sequential pretreatment procedures are degreasing in HCl at 1:1 concentration at 25 °C for 5 s and then rinsing three times in deionized water for 5 s in each rinse in order to remove any traces of HCl from the samples.

The aluminum alloys were anodized using the following bath solutions:

○ Citric acid concentration [1 M];
○ As a reference, use a sulfuric acid solution [1 M];
○ Citric acid concentration [1 M] + 5 mL/L of sulfuric acid; finally
○ Citric acid concentration [1 M] + 10 mL/L of sulfuric acid.

A lead bar served as the cathode, and samples of the aluminum alloys 2024-T3, 6061-T6, and 7075-T6 served as the anodes. Four different electrolyte solutions, current densities that anodized (i) of 3 and 4.5 A/dm^2^, and a DC power supply model XLN30052-GL (AC, USA) were the conditions used throughout the process of anodizing [43]. An hour-long anodization took place in a bath of cold water at 0 °C ± 2 °C with the solution being constantly stirred. Temperature variation is crucial for type III hard anodizing; thus, it needs to be managed and kept to a minimum since too much electricity generates heat, which can dissolve the film entirely or partially [44]. Once the pieces had been anodized, after they were washed with deionized water, a sealing process was carried out, which involved immersing them in 95 ± 4 °C deionized water for an hour. In addition to improving abrasion resistance and reducing or eliminating porosity in the anodizing, the hot-water sealing process also lessens the deteriorating phenomenon known as efflorescence, which is caused by aluminum exposed to the outside [45,46]. Hydration of the anodic coating is believed to be the sealing procedure that takes place in boiling water. Aluminum hydroxide is created when aluminum oxide and water combine, filling the pores of the coating and sealing the surface by the reaction presented in Equation (1) [47]:

(1) $${\mathrm{Al}}_{2}O_{3}+\left(n+1\right)H_{2}O\to 2\mathrm{AlOOH}+nH_{2}O\;\;\;\;\;\;\;\;\;\;\left(n>1\right)$$

**Figure 1:**
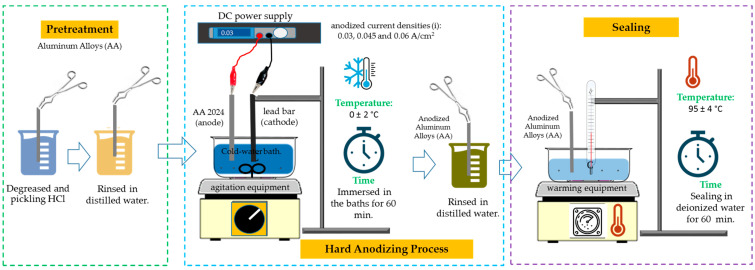
Schematic of the anodizing process used on aluminum alloys 2024-T6, 6061-T6, and 7075-T6.

The anodic surface gains greater uniformity and flatness because of this procedure. The objective of this water-sealing method is to protect the substrate from environmental degradation by sealing the porous coatings. Table 1 lists the parameters of the anodizing procedure used in this study. The anodizing procedure employed in this work is shown in Figure 1.

### 2.3. Microstructural Characterization

Utilizing Zeiss model Sigma 300 VP (Oberkochen, Baden-Wurtemberg, Germany) equipment, scanning electron microscopy (SEM) at 500× magnification was used to obtain surface and cross-sectional morphology. Five measurements per sample were performed with the computer software to determine the thickness and microstructure in cross-section. The morphology was examined using backscattered electrons (BSEs) at a beam energy of 20 kV, and the chemical makeup of the cross-sections was ascertained employing dispersive energy X-ray spectroscopy (EDS).

### 2.4. Vickers Hardness Test

The cross-section of the anodized specimen was assessed for Vickers hardness using a microhardness tester (Wilson Tester 402 MVD Lake Bluff, IL, USA); 15 readings per sample were obtained with a 0.05 gf load and a 15 s dwell length in compliance with ASTM E92 [48].

### 2.5. Corrosion Test

Corrosion studies were conducted using three-electrode cells. The working electrodes were anodized materials, a platinum mesh counter electrode, and a saturated calomel electrode as reference (SCE). The ZRA Solartron 1287A (Bognor Regis, Arun, West Sussex, UK) potentiostat and galvanostat were utilized. Every test was run in duplicate and involved dipping in a 3.5 wt.% NaCl solution at ambient temperature [49,50,51]. Utilizing cyclic potentiodynamic polarization curve testing (CPPC), the corrosion resistance of the anodized components was evaluated. In accordance with ASTM G61-11 [52], using a potential sweep from −0.3 to 1.0 V of OCP, a scan rate of 0.06 V/min, and a full polarization cycle, the CPPC was performed. By following the ASTM G106-15 standard [53]., electrochemical impedance spectroscopy (EIS) tested a frequency range of 0.01 to 100,000 Hz, applied a 10 mV RMS amplitude, and obtained 35 points per decade Utilizing ”Zview-4” software (https://www.scribner.com/software/68-general-electrochemistr376-zview-for-windows/ Scribner Associates, accessed on 15 June 2024, Inc. por Berek Johnson, Southern Pines, NC, USA), the EIS spectra were examined in terms of electrical equivalent circuits.

## 3. Results

### 3.1. Chemical Composition by X-ray Fluorescence (XRF)

The chemical composition of the aluminum alloy was determined by utilizing X-ray fluorescence (see Table 2). It is important to observe the presence of Cu, Mg, Si, Zn, and Fe in all three alloys; the high content of Cu, Mg, and Si in the 2024-T3 alloy; the high content of Mg, Si, and Fe in the 6061-T6 alloy; and the higher amount of Zn, Mg, and Cu in the 7075-T6 alloy. In all three aluminum alloys, Cr, Mn, and Ti were detected in lower amounts than the main alloying elements for each alloy. In the three alloys used, the chemical composition of the aluminum alloys is in accordance with what has been proposed by other authors [54].

### 3.2. Microstructural Characterization (SEM)

#### 3.2.1. Surface Morphology

**Figure 2:**
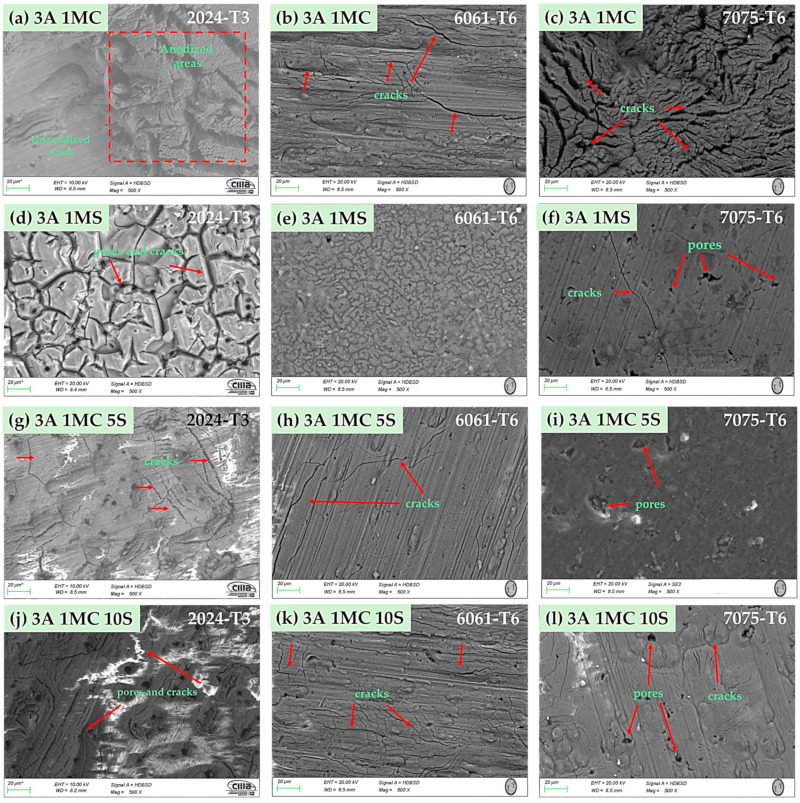
Surface morphology of several aluminum alloys anodized at 3 A/dm^2^ in various bath solutions as determined by SEM-BSE. (**a**) 3A 1MC, 2024-T3; (**b**) 3A 1MC, 6061-T6; (**c**) 3A 1MC, 7075-T6; (**d**) 3A 1MS, 2024-T3; (**e**) 3A 1MS, 6061-T6; (**f**) 3A 1MS, 7075-T6; (**g**) 3A 1MC 5S, 2024-T3; (**h**) 3A 1MC 5S, 6061-T6; (**i**) 3A 1MC 5S, 7075-T6; (**j**) 3A 1MC 10S, 2024-T3; (**k**) 3A 1MC 10S, 6061-T6; (**l**) 3A 1MC 10S, 7075-T6.

**Figure 3:**
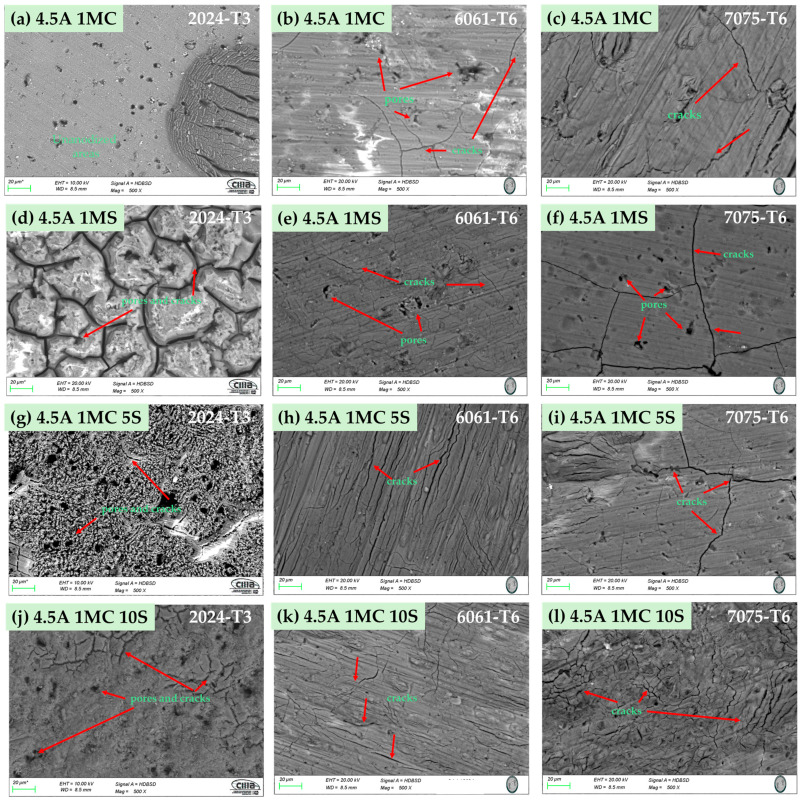
Surface morphology of several aluminum alloys anodized at 4.5 A/dm^2^ in various bath solutions as determined by SEM-BSE. (**a**) 4.5A 1MC, 2024-T3; (**b**) 4.5A 1MC, 6061-T6; (**c**) 4.5A 1MC, 7075-T6; (**d**) 4.5A 1MS, 2024-T3; (**e**) 4.5A 1MS, 6061-T6; (**f**) 4.5A 1MS, 7075-T6; (**g**) 4.5A 1MC 5S, 2024-T3; (**h**) 4.5A 1MC 5S, 6061-T6; (**i**) 4.5A 1MC 5S, 7075-T6; (**j**) 4.5A 1MC 10S, 2024-T3; (**k**) 4.5A 1MC 10S, 6061-T6; (**l**) 4.5A 1MC 10S, 7075-T6.

The surface morphology of the anodized samples with varying current densities of 3 and 4.5 A/dm^2^ in the bath solutions is displayed by SEM in Figure 2 and Figure 3. All anodized samples of alloys 2024-T6, 6061-T6, and 7075-T6, with the two anodizing current densities of 3 and 4.5 A/dm^2^, revealed porosity and surface cracking (marked with red arrows) on the coating. This behavior was consistent across all alloys, regardless of the anodizing bath solution utilized. Alloy 2024-T3 samples anodized in a 1 M citric acid solution show the presence of zones devoid of anodized coating [Figure 2a, red box, and Figure 3a].

Samples of alloy 2024-T3 anodized in 1 M H_2_SO_4_ solution with current densities of 3 and 4.5 A/dm^2^ showed a surface morphology known as “dry mud or soil” [Figure 2d and Figure 3d]. The same surface morphology was also present in the 6061-T6 aluminum sample anodized in sulfuric acid with 3 A/dm^2^ (Figure 2e). Other authors have described this type of morphology [55,56]. The samples that were anodized in mixtures of sulfuric and citric acids with different current densities showed surface cracking and porosity, yet the coatings are often continuous throughout the whole surface.

#### 3.2.2. Morphology of Cross-Section

**Figure 4:**
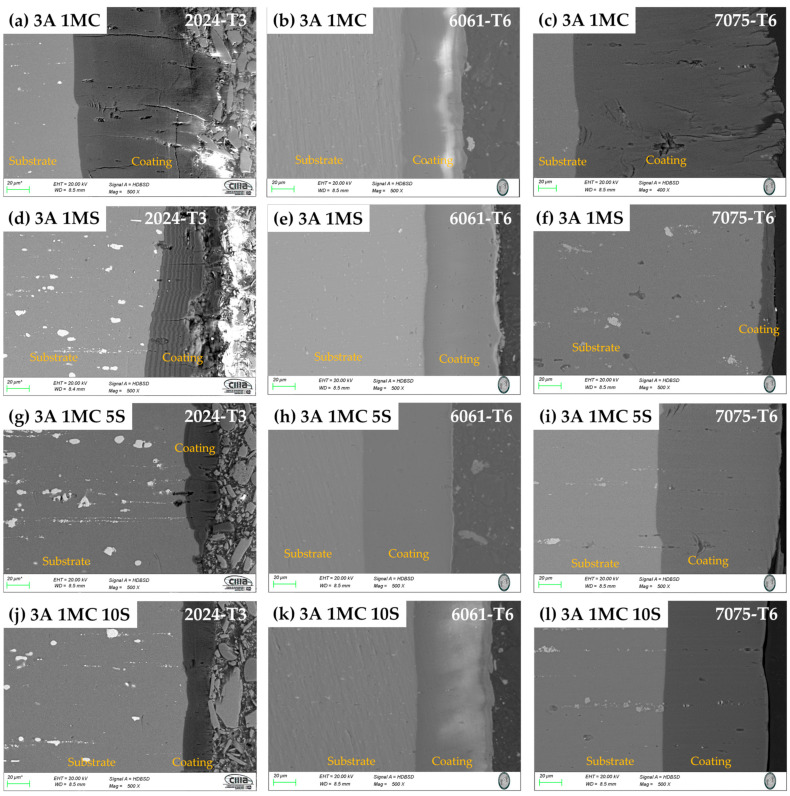
Cross-section by SEM of different aluminum alloys anodized at 3 A/dm^2^ in different bath solutions. (**a**) 3A 1MC, 2024-T3; (**b**) 3A 1MC, 6061-T6; (**c**) 3A 1MC, 7075-T6; (**d**) 3A 1MS, 2024-T3; (**e**) 3A 1MS, 6061-T6; (**f**) 3A 1MS, 7075-T6; (**g**) 3A 1MC 5S, 2024-T3; (**h**) 3A 1MC 5S, 6061-T6; (**i**) 3A 1MC 5S, 7075-T6; (**j**) 3A 1MC 10S, 2024-T3; (**k**) 3A 1MC 10S, 6061-T6; (**l**) 3A 1MC 10S, 7075-T6.

**Figure 5:**
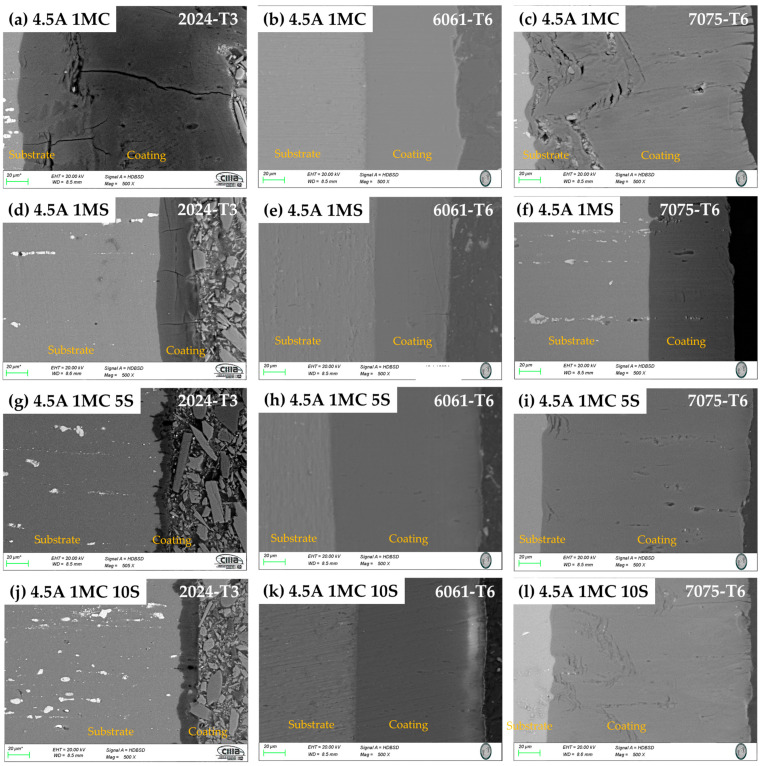
Cross-section by SEM of different aluminum alloys anodized at 4.5 A/dm^2^ in different bath solutions. (**a**) 4.5A 1MC, 2024-T3; (**b**) 3A 1MC, 6061-T6; (**c**) 4.5A 1MC, 7075-T6; (**d**) 4.5A 1MS, 2024-T3; (**e**) 4.5A 1MS, 6061-T6; (**f**) 4.5A 1MS, 7075-T6; (**g**) 4.5A 1MC 5S, 2024-T3; (**h**) 4.5A 1MC 5S, 6061-T6; (**i**) 4.5A 1MC 5S, 7075-T6; (**j**) 4.5A 1MC 10S, 2024-T3; (**k**) 4.5A 1MC 10S, 6061-T6; (**l**) 4.5A 1MC 10S, 7075-T6.

SEM cross-sectional micrographs of the samples anodized in the various solutions are displayed in Figure 4 and Figure 5. In these coatings, porosity and cracks can sometimes penetrate through to the base material from the coating’s surface. For the alloys 2024-T3 and 7075-T6, the samples anodized with 3 and 4.5 A/dm^2^ also exhibit this same breaking behavior, which extends from the coating’s surface to the base metal. In all anodizing solutions, the anodized alloy 2024-T3 samples with the two current densities displayed more cracking. The 2024-T6 alloy samples (Figure 4a,d,g,j and Figure 5a,d,g,j) showed signs of porosity and cracking, which can occasionally seep through the coating surface and into the substrate. When anodized in 1MC 5S and 1MC 10S solutions at the two anodizing current densities utilized, alloy 7075-T6 did not exhibit any cracking (Figure 4i,l). The 1MC and 1MS solution anodized samples for the 7075-T6 alloy did exhibit porosity and cracking [Figure 4c,f and Figure 5c,f]. In all solutions and anodizing current densities, the surface of the anodized 6061-T6 alloy samples showed no signs of porosity or cracking [Figure 4b,e,h,k, and Figure 5b,e,h,k]. These same figures demonstrate that these coatings do not exhibit cracking and are more homogeneous than the other alloys.

#### 3.2.3. Chemical Composition of the Cross-Section by SEM-EDS

**Figure 6:**
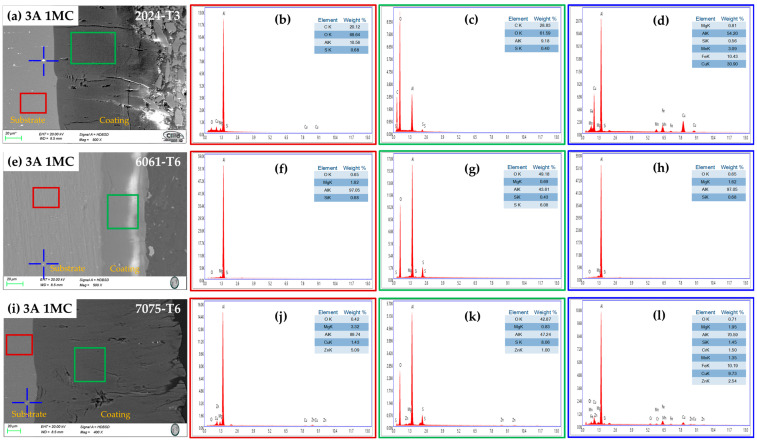
Aluminum alloys anodized at 3 A/dm^2^ in a bath solution [1 M] of citric acid: cross-section morphology by SEM. (**a**) 3A 1MC, 2024-73; (**e**) 3A 1MC, 6061-T6 2024-T3; and (**i**) 3A 1MC, 7075-T6 chemical composition determined by EDS spectra (marked in the morphology in blue cross and red and green). (**b**–**d**) 3A 1MC,2024-73; (**f**–**h**) 3A 1MC, 6061-T6 2024-T3; and (**j**–**l**) 3A 1MC, 7075-T6.

**Figure 7:**
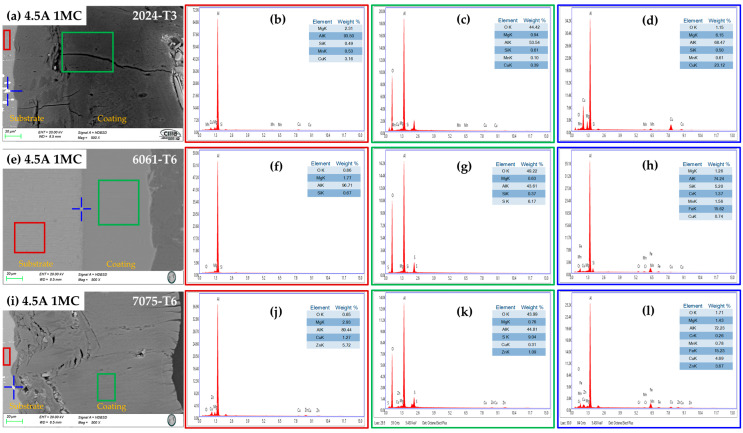
Aluminum alloys anodized at 4.5 A/dm^2^ in a bath solution [1 M] of citric acid: cross-section morphology by SEM (**a**) 4.5A 1MC, 2024-73; (**e**) 4.5A 1MC, 6061-T6 2024-T3; and (**i**) 4.5A 1MC, 7075-T6 chemical composition determined by EDS spectra (marked in the morphology in blue cross and red and green). (**b**–**d**) 4.5A 1MC,2024-73; (**f**–**h**) 4.5A 1MC, 6061-T6 2024-T3; and (**j**–**l**) 4.5A 1MC, 7075-T6.

Figure 6 and Figure 7 show the cross-section micrographs produced by SEM and the chemical compositions determined by EDS of the anodized aluminum alloys under different conditions. Figure 6a,e,i and Figure 7a,e,i, indicate the layer of anodized coating produced in the [1 M] citric acid solution and the alloys used. The second column (red boxes) represents the elements obtained by EDS of the base material, where it is evident that Al and Cu compose the majority of the 2024-T3 alloy (Figure 6b and Figure 7b); the 6061-T6 alloy presents the elements Al, Si, and Mg (Figure 6f and Figure 7f); and the 7075-T6 alloy presents the elements Al, Zn, Mg, and Cu (Figure 6j and Figure 7j) corresponding to each type of aluminum alloy. The third column (green boxes) shows the coatings’ chemical composition formed during the anodizing process of the different alloys, where high concentrations of oxygen can be observed in all cases, corresponding to the creation of the aluminum oxide layer (alumina Al_2_O_3_) in the anodizing process. Additionally, it can be observed in these figures that some elements that make up the substrate are also part of the coating composition, such as Cu, Si, Mg, and Zn [Figure 6c,g,k (green box) and Figure 7c,g,k (green box)]. The blue boxes in the fourth column of Figure 6 and Figure 7 represent the chemical composition of the precipitates of every alloy that is indicated in the original image by a blue cross. The composition of alloy 2024-T3, intermetallic compounds, is displayed in Figure 6d (blue cross), which has high contents of Cu, Fe, Mn, and Mg, which form the precipitates S (Al_2_CuMg) and θ (Al_2_Cu). In the 6061-T6 aluminum alloy, the Fe, Mg, and Si elements are observed; these elements form the intermetallic precipitates AlSiFe (Figure 6h and Figure 7h (blue cross)). In the 7075-T6 aluminum alloy, the precipitates contain the elements Fe, Cu, Zn, Mg, and Mn, which form the AlCuMg phases.

### 3.3. Thickness of Anodized Aluminum Alloys

**Figure 8:**
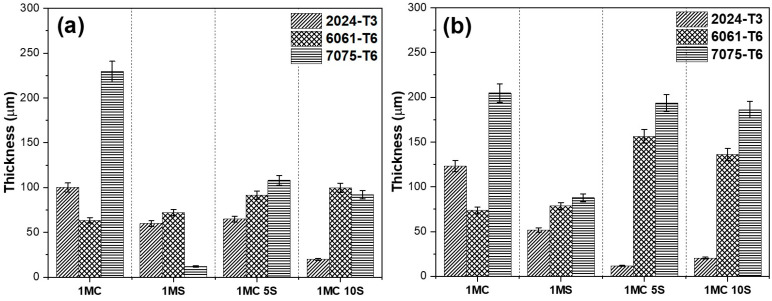
Different aluminum alloy coating thicknesses anodized in various solutions and current densities: (**a**) 3 A/dm^2^ and (**b**) 4.5 A/dm^2^.

The thickness measured by SEM in cross-sectional micrographs of samples anodized in various alloys, with varying bath solutions consisting of sulfuric and citric acid, and current densities of 3 and 4.5 A/dm^2^, is displayed in Figure 8. Figure 8a shows that in most of the solutions, the aluminum alloy 7075-T6 had the highest oxide thicknesses generated during the anodizing process, whereas the aluminum alloys 6061-T6 and 2024-T3 had the lowest thickness values. Figure 8b shows practically the same effect, with the lowest thickness for the 2024-T3 alloys, followed by the 6061-T6 alloy and finally the 7075-T6 aluminum alloy, with the highest thickness. For all three alloys, the thickness of the oxide formed increases with increasing anodizing current density. The 2024-T3 and 6061-T6 alloys’ intermetallic phases prevent oxide growth, which is why the oxide thickness in the 6061-T6 and 7075-T6 alloys increases with a current density of 4.5 A/dm^2^ while the oxide thickness in the 2024-T3 alloy decreases as a result of the oxide dissolving from the high anodizing current. When an addition such as citric acid prevents the oxide coating from dissolving, thin coatings are created [57]. The oxide thickness produced on the three aluminum alloys grows from 5 to 10 mL/L as the sulfuric acid concentration rises, improving the electrolytes’ conductivity.

### 3.4. Vickers Microhardness

**Figure 9:**
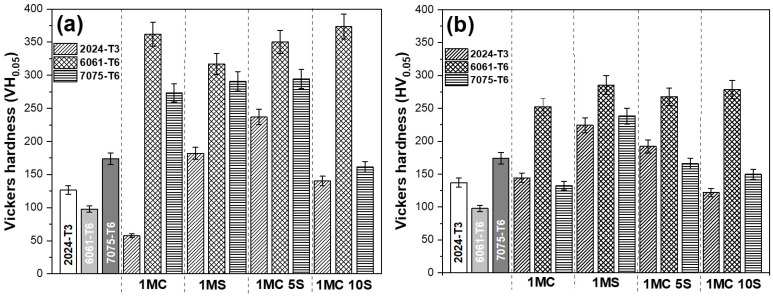
Vickers microhardness of different aluminum alloys anodized in diverse solutions and current densities: (**a**) 3 A/dm^2^ and (**b**) 4.5 A/dm^2^.

The results of Vickers microhardness studies of the various alloys anodized with current densities of 3 and 4.5 A/dm^2^ and bath solutions are shown in Figure 9. In this study, all anodized samples exhibited a microhardness greater than the non-anodized material, regardless of the solution or anodized current density used. Only the samples 2024-T3 and 7075-T6, anodized in citric acid with current densities of 3 A/dm^2^ and 4.5 A/dm^2^, respectively, did not reach hardnesses higher than the non-anodized materials. Because of the small imperfections created by the fine precipitates, the oxide layer that forms on 6061-T6 Al alloy has the best microhardness. On the other hand, because of significant flaws created by coarse intermetallic phases, aluminum alloy 7075-T6 has a lower microhardness than the 6061-T6 Al alloy. Compared with the 6061-T6 and 7075-T6 Al alloys, the oxide layer generated on the 2024-T3 Al alloy has a lower microhardness. This is attributed to significant flaws and fissures in the oxide layer brought on by oxygen production and the high reactivity of copper-rich intermetallic phases. In this work, the highest Vickers microhardness was obtained in the 6061-T6 alloy in the different solutions anodized at 3 A/dm^2^ with values above 300 HV, which is a typical value for hard anodizing [58].

### 3.5. Electrochemical Corrosion Test

#### 3.5.1. Cyclic Potentiodynamic Polarization Curve (CPPC)

**Figure 10:**
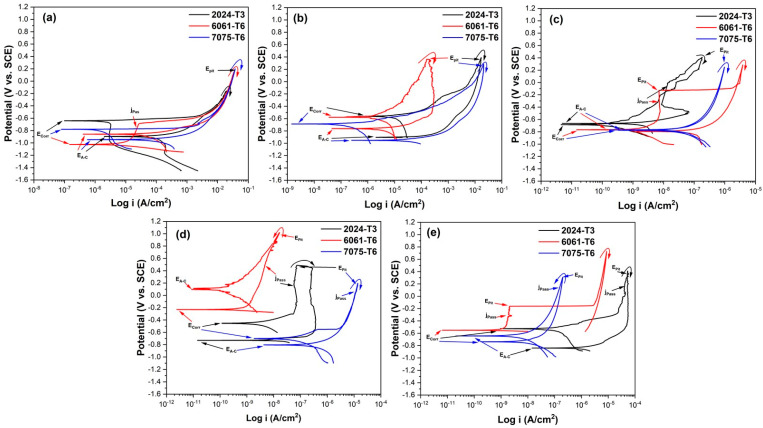
CPPC exposed to the 3.5 wt.% NaCl solution of different aluminum alloys anodized in current density of 3 A/dm^2^: (**a**) Non-anodized material; (**b**) 1MC; (**c**) 1MS; (**d**) 1MS 5S, and (**e**) 1MC 10S.

The results of CPPC measurements of several alloys with various bath solutions that were anodized with a current density of 3 A/dm^2^ and subjected to a 3.5 wt.% NaCl solution are displayed in Figure 10. A CPPC is typically utilized to examine a material’s propensity for pitting corrosion within a certain corrosion environment. The CPPC, which provides information on the anodic, cathodic, and hysteresis ranches of the anodized samples on the corrosion phenomena, was used to analyze the corrosion process. Anodized samples with nobler E_corr_ values are less susceptible to corrosion [59,60]. The pitting potential (E_pit_) is the potential value at which the current rises and the pitting assault occurs. The aluminum is more resistant to pitting the higher its E_pit_ value is. The material’s propensity to develop pits is measured by the difference between E_pit_ and E_corr_; large differences suggest that the material’s resistance to pitting corrosion has improved [61,62].

The anodic to cathodic transition potential (E_A-C_) is defined as the potential required when the anodic current density varies with the cathodic current density. The passive film’s persistence is ascertained by comparing the E_A-C_ and E_corr_. If there is positive hysteresis, the passive layer will not be stable if the E_A-C_ is nobler than the E_corr_. If the E_corr_ is more noble than the E_A-C_, the passive layer will endure and exhibit positive hysteresis [63]. In this way, the potential for E_A-C_ is more negative than that for E_corr_ in the reversal curves of the anodized materials in the various solutions. In the potential sweep, the lack of a hysteresis loop for these samples could suggest broad corrosion and an active surface rather than the absence of localized corrosion. Pitting and crevice corrosion are associated with positive hysteresis, and general corrosion is associated with negative hysteresis [64].

**Figure 11:**
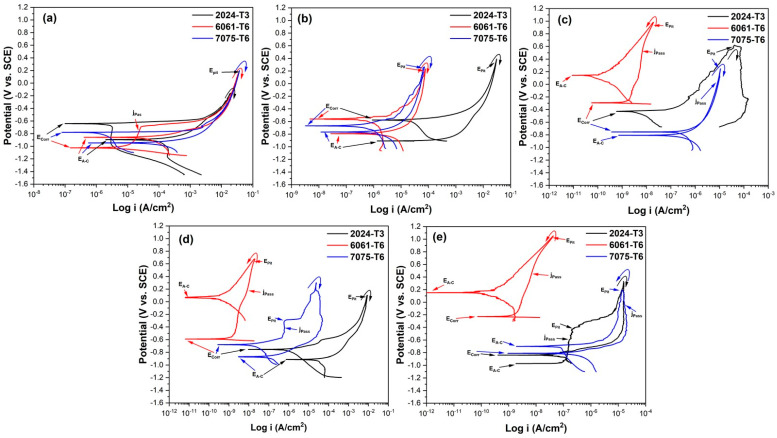
CPPC of different aluminum alloys anodized in current density of 4.5 A/dm^2^: (**a**) Non-anodized material; (**b**) 1MC; (**c**) 1MS; (**d**) 1MS 5S; and (**e**) 1MC 10S.

Figure 10a and Figure 11a show the materials without anodizing. The material that presented the noblest E_corr_ was aluminum 2024-T3, followed by 7075-T6, and finally, the 6061-T6 alloy. As for the E_A-C_, all the different aluminum alloys presented very similar values, from −0.858 to −0.945 V vs. SCE. The alloys 2024-T3 and 7075-T6 did not present j_pass_ values; only alloy 6061-T6 presented a j_pass_ value of 2.60 × 10^−5^ A/cm^2^. For corrosion current density (j_corr_), alloy 6061-T6 presented the highest value, while alloys 2024-T3 and 7075-T6 presented similar values of 0.30 × 10^−6^ A/cm^2^, which indicates that these two materials are less susceptible to corrosion out of the three alloys evaluated in this work. Regarding the type of hysteresis for the materials without anodizing, all of them are positive, which indicates a localized corrosion type. For the alloys anodized in the 1MC solution (Figure 10b), the most noble E_corr_ was present in alloys 2024-T3 and 6061-T6, while alloy 7075-T6 presented the less noble E_corr_. In the case of E_A-C_, all the alloys presented potentials more negative than their corresponding E_corr_, which is indicative that the oxide formed during anodizing is a stable and protective oxide. None of the three alloys presented j_pass_, which indicates that all of them are in activation during the whole anodic branch. The highest j_corr_ occurred in alloy 6061-T3, followed by alloy 2024-T3, and finally, alloy 7075-T6, with values of 8.02 × 10^−6^, 1.50 × 10^−6^, and 0.11 × 10^−6^ A/cm^2^, respectively. Since the hysteresis type was positive, the material anodized in citric acid solution also exhibits localized corrosion [1 M].

The sulfuric acid anodized alloys that were used as reference are shown in Figure 10c. In this case, the E_corr_ are very similar as are the E_A-C_; this shows that the oxide produced during the anodizing procedure is stable and protective, as expected. The j_pass_ presented higher values for alloy 7075-T6, followed by alloy 2024-T3, and finally, alloy 6063-T6; this same behavior was presented in the j_corr_, which indicates that in this anodizing solution and with a current density of 3 A/dm^2^, the alloy with the lowest corrosion resistance is alloy 7075-T6, followed by alloy 2024-T3, and finally, alloy 6063-T6, with j_corr_ values of 0.0296 × 10^−6^, 0.0296 × 10^−6^, and 0.0296 × 10^−6^ A/cm^2^, respectively. The type of hysteresis was positive, indicating localized corrosion. For the 1MC 5S solution anodized alloys (Figure 10d), the most noble E_corr_ was presented in the 6061-T6 alloys, and then the 2024-T3 alloy, while the 7075-T6 alloy presented less noble E_corr_. The behavior of E_A-C_ and E_pit_ was like that of E_corr_. As for j_pass_, the lowest value was found in alloy 6061-T6 (0.0004 × 10^−5^ A/cm^2^), followed by alloy 7075-T6 (0.861 × 10^−5^ A/cm^2^), and finally, alloy 2024-T3 (3.97 × 10^−5^ A/cm^2^). The j_corr_ was lowest in the 6061-T6 alloy, followed by the 2024-T3 alloy, and finally, the 7075-T6 alloy. In this solution, negative hysteresis was presented in alloy 6061-T6, indicating uniform corrosion in this alloy, and positive hysteresis was presented for alloys 2024-T3 and 7075-T6, indicating localized corrosion. These results indicate that alloy 6061-T6 presents better corrosion resistance when anodized in a 1MC 5S solution and a current density of 3 A/dm^2^. The alloys anodized in a solution of 1MC 10S (Figure 10e) showed the most noble E_corr_ in alloy 2024-T3 and then alloy 6061-T6; finally, alloy 7075-T6 showed the least noble E_corr_. In alloys 2024-T3 and 6061-T6, the E_A-C_ value was more negative than E_corr_, which indicates that the oxide formed during anodizing is stable and will persist. As for the j_pass_, the lowest value was presented in alloy 6061-T6 (0.0002 × 10^−5^ A/cm^2^), followed by alloy 7075-T6 (0.014 × 10^−5^ A/cm^2^), and finally, alloy 2024-T3 (4.09 × 10^−5^ A/cm^2^). The j_corr_ was lower in alloy 6061-T6, followed by alloy 7075-T6, and finally, alloy 2024-T3, presenting values of 0.0010 × 10^−6^, 0.0046 × 10^−6^, and 0.104 × 10^−6^ A/cm^2^, respectively. In this solution, negative hysteresis was presented in alloy 7075-T6, indicating uniform corrosion, and positive hysteresis for alloys 2024-T3 and 6061-T6, indicating localized corrosion. For this anodizing condition, alloy 6061-T6 is again the alloy with the best corrosion resistance. Lower corrosion susceptibility is indicated by the anodized samples’ nobler E_corr_ values [65,66].

When comparing all the anodizing solutions at the anodizing current density of 3 A/dm^2^, the samples that presented better corrosion resistance in the CPPC were the 6061-T6 alloy anodized in solutions of 1MC 5S and 1MC 10S, which have better corrosion resistance than conventional anodizing. All the electrochemical data from the CPPC subjected to a 3.5 wt.% NaCl solution, anodizing in various baths, and current density of 3 A/dm^2^ are displayed in Table 3.

Figure 11 shows the results obtained from CPPC measurements of the different alloys with different bath solutions and anodized with a current density of 4.5 A/dm^2^, exposed to the 3.5 wt.% NaCl solution. For the 1MC solution anodized alloys (Figure 11b), the E_corr_ is very similar for all the aluminum alloys; in this case, the most noble E_corr_ was present in alloys 2024-T3 and 6061-T6, while alloy 7075-T6 presented a less noble E_corr_. In the case of E_A-C_, all the alloys showed more negative potentials than their corresponding E_corr_, which is indicative that the oxide formed during anodizing will persist as a protective oxide. None of the three alloys presented j_pass_, which indicates that all of them are in activation during the whole anodic branch. The highest j_corr_ was found for alloy 2024-T3, followed by alloy 7075-T6, and finally, alloy 6061-T3, with values of 48.3 × 10^−6^, 0.51 × 10^−6^, and 0.20 × 10^−6^ A/cm^2^, respectively. The positive hysteresis type is associated with a reduction in passivity as a result of pitting and crevice corrosion. Figure 11c shows the sulfuric acid anodized alloys that were used as reference. In this case, the E_corr_ values are very varied, with the most noble potential in alloy 6061-T6 (−0.293 V vs. SCE) followed by alloy 2024-T3 (−0.431 V vs. SCE) and the least noble potential in alloy 7075-T6 (−0.754 V vs. SCE). In alloy 2024-T3 there was no E_A-C_, and its hysteresis cycle was positive and very large, which means a greater rupture of the oxide film; this makes it difficult to re-establish passivity. For the 6061-T6 alloy, E_A-C_ was nobler than E_corr_, so the passive layer will not be stable at E_corr_. In aluminum alloy 7075-T6, the difference between E_corr_ and E_A-C_ was very small; therefore, uniform or widespread corrosion occurs. The 2024-T3 alloy did not show any j_pass_ as it is in activation during the whole anodic branch, while the highest j_pass_ was present in the 7075-T6 alloy, followed by the 6061-T6 alloy. For the j_corr_, the 7075-T6 alloy had the lowest value, followed by the 2024-T3 alloy, and finally, the 6061-T6 alloy. The type of hysteresis is positive for alloys 2024-T3 and 7075-T6, while alloy 6061-T6 showed negative hysteresis related to generalized corrosion. This indicates that the material with the best anodized corrosion resistance in this solution [1 M] sulfuric acid is alloy 6061-T6.

The alloys anodized in solution 1MC 5S are presented in Figure 11d. The most noble E_corr_ was found in alloy 6061-T6, followed by alloy 7075-T6, while alloy 2024-T3 showed the least noble E_corr_. The behavior of E_pit_ and E_A-C_ was like that of E_corr_. The lowest value of j_pass_ was found in alloy 6061-T6 (0.00040 × 10^−5^ A/cm^2^), followed by alloy 2024-T3 (0.012 × 10^−5^ A/cm^2^), and finally, alloy 7075-T6 (0.059 × 10^−5^ A/cm^2^). The j_corr_ was lowest in alloy 6061-T6, followed by alloy 7075-T6, and finally, alloy 2024-T3. In this solution, positive hysteresis was present for alloys 2024-T3 and 7075-T6, indicating localized or crevice corrosion, while alloy 6061-T6 presented negative hysteresis, indicating uniform corrosion. These results indicate that alloys 6061-T6 and 7075-T6 show better corrosion resistance when anodized in citric acid solution [1 M] with the addition of 5 mL/L sulfuric acid. Finally, the samples anodized with citric acid solution [1 M] and 10 mL/L sulfuric acid, with a current density of 4.5 A/dm^2^ and immersed in 3.5 wt.% NaCl solution, are presented in Figure 11e. Again, the 6061-T6 alloy presented the noblest E_corr_ value, followed by the 7075-T6 alloy and finally, the 2024-T3 alloy. Regarding the E_A-C_ potential, the alloys 2024-T3 and 6061-T6 did not present much difference with respect to E_corr_; therefore, in these two alloys, a uniform or generalized type of corrosion will occur. In the aluminum 6061-T6 alloy, the E_corr_ and E_A-C_ are very different, so localized or crevice corrosion will occur. The lowest j_pass_ value was found in alloy 6061-T6 (0.00053 × 10^−5^ A/cm^2^), followed by alloy 7075-T6 (1.03 × 10^−5^ A/cm^2^), and finally, alloy 2024-T3 (1.57 × 10^−5^ A/cm^2^). The lowest j_corr_ was obtained for alloy 6061-T6, followed by alloy 7075-T6, and finally, alloy 2024-T3, with values of 0.0011 × 10^−6^, 0.168 × 10^−6^, and 0.297 × 10^−6^ A/cm^2^, respectively. All three of the study alloys had negative hysteresis under these anodizing settings, indicating that generalized corrosion will happen and that surface passivation will be stronger at higher potentials. This phenomenon, also noted in the AA7075 alloys anodized with different baths, is caused by the protective layer that forms a barrier during the anodizing process [67].

The 6061-T6 alloys fabricated in the 1MC 5S and 1MC 10S solutions, which are more resistant to corrosion than traditional sulfuric acid anodizing, demonstrated better corrosion resistance in CPPC when comparing all anodizing solutions at an anodizing current density of 4.5 A/dm^2^. Regardless of the alloy type, all of the samples anodized in citric acid solutions and in mixes of citric and sulfuric acid showed higher corrosion resistance than the non-anodized material. The alloy 6061-T6 showed the greatest resistance to corrosion, followed by the alloys 7075-T6 and 2024-T3. Table 4 shows all of the electrochemical data from the CPPCs that were anodized in different baths, exposed to a 3.5 wt.% NaCl solution, and had a current density of 4.5 A/dm^2^.

#### 3.5.2. Electrochemical Impedance Spectroscopy (EIS)

**Figure 12:**
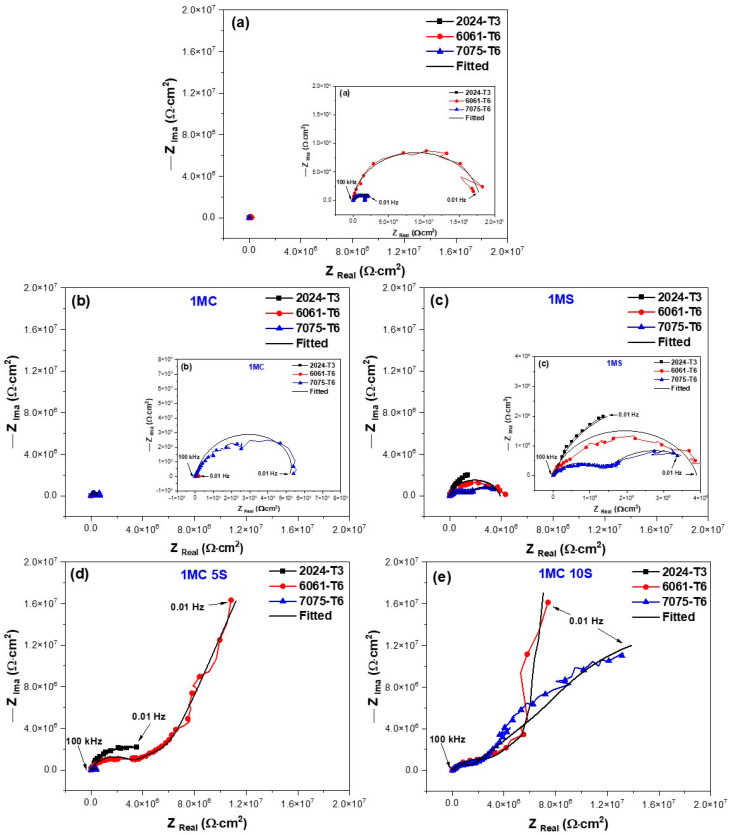
Nyquist plots of different aluminum alloys evaluated in 3.5 wt.% NaCl solution and anodized in current density of 3 A/dm^2^ and different solutions: (**a**) non-anodized material; (**b**) 1MC; (**c**) 1MS; (**d**) 1MS 5S; and (**e**) 1MC 10S.

**Figure 13:**
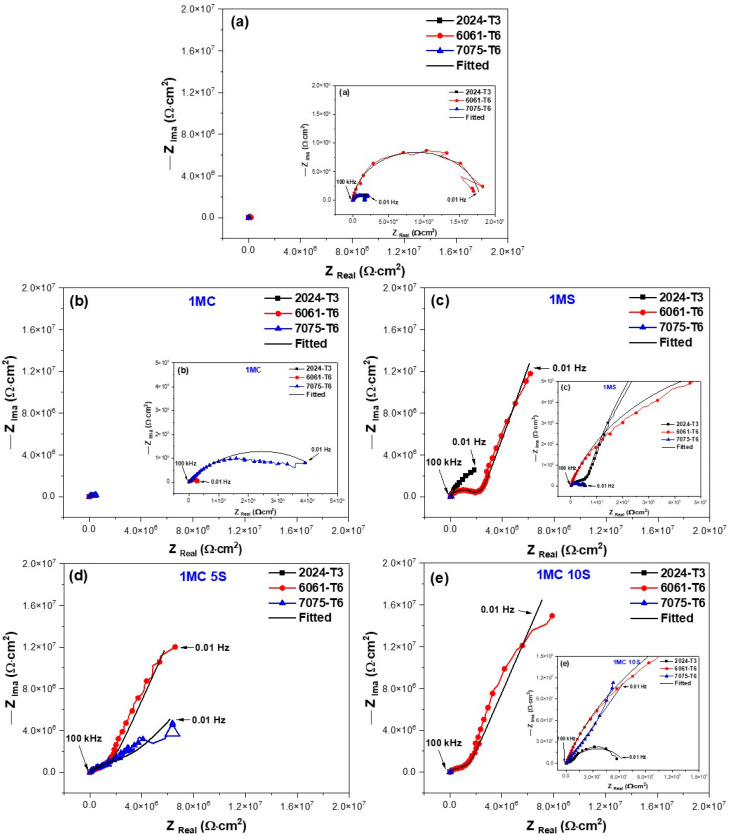
Nyquist plots of different aluminum alloys evaluated in 3.5 wt.% NaCl solution and anodized in current density of 4.5 A/dm^2^ and different solutions: (**a**) non-anodized material; (**b**) 1MC; (**c**) 1MS; (**d**) 1MS 5S; and (**e**) 1MC 10S.

The characteristics of the EIS spectra of the samples analyzed in this study are shown in Figure 12 and Figure 13. To give a more complete investigation of the corrosion susceptibility and protective qualities of the anodized materials, EIS analysis was carried out in addition to CPPC. Figure 12 shows the Nyquist curve generated for the anodized and non-anodized samples exposed to 3.5 wt.% NaCl solution at a current density of 3 A/dm^2^. In the case of alloys 2024-T3, 6061-T6, and 7075-T6, which have not had anodizing treatment, the typical behavior of an oxide layer that forms naturally on the surface of aluminum alloys is shown in Figure 12a and Figure 13a. This behavior might be due to the related circuits’ oxide layer having a combination of resistive and constant phase elements [68,69,70]. The majority of the alloys employed in this work exhibit a high-frequency semicircle, capacitive behavior at lower frequencies for anodizing with the various citric–sulfuric acid mixes, and anodized current densities of 3 and 4.5 A/dm^2^ (Figure 12b–e). These behaviors follow the barrier layer’s and porous layer’s respective properties. The depressed semicircles at high frequency (1 × 10^2^–1 × 10^4^ Hz) of the samples anodized in citric acid solutions exhibit a lower corrosion rate than the non-anodized aluminum alloy, as demonstrated by the Nyquist plot in Figure 12b,d,e. A line with a 45° slope at the lowest frequencies indicates diffusion phenomena, indicating the characteristics of the barrier layer, and only a few samples of aluminum 6061-T6 and 7075-T6 aluminum showed a capacitive arc at high frequencies, corresponding to the characteristics of the porous layer (Figure 12d,e). The vertical slope results from Warburg diffusion, and the semicircles’ diameter represents the charge transfer resistance (R_ct_) [71,72]. The main relationship between the two kinetic processes of the Nyquist diagrams (Figure 12d,e) with the two time constants present is the semi-passivation that results from the corrosion products created filling the pores and cracks and diffusing toward the inside of the porous layer. The re-passivation of the coating’s porous layer corresponds to the second time constant.

The Nyquist diagrams obtained at 4.5 A/dm^2^ for all samples of aluminum alloys following exposure to a 3.5 wt.% NaCl solution are displayed in Figure 13. Most of the aluminum alloys used in this work displayed two capacitive semicircles, one at high frequencies corresponding to the characteristics of the porous layer and the other at high frequencies giving the characteristics of the barrier layer, both formed during the anodizing process. This is similar to the anodizing current density of 3 A/dm^2^. Figure 13a–e shows this. Also, at this anodizing current density of 4.5 A/dm^2^, some samples showed a 45° slope corresponding to diffusion phenomena in both the porous and barrier layers of the material; in most cases, this phenomenon occurred in the alloy 6061-T6 and 7075-T6 in anodizing solutions of 1MC 5S and 1MC 10S (Figure 13d,e and zoom in Figure 13e). It indicates that this coating has a greater impedance than all the other coatings in the tested frequency range. Although the 6061-T6 aluminum sample anodized in sulfuric acid exhibits the same phenomena, this material is employed as a reference in this work (Figure 13c).

**Figure 14:**
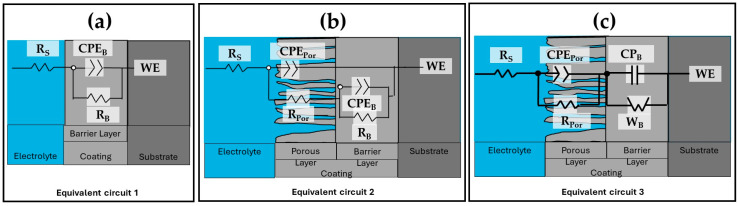
Equivalent electric circuits: (**a**) non-anodized alloys, (**b**) some anodized alloys, and (**c**) alloys with Warburg element (W_B_).

The equivalent electrical circuits (EECs) that were proposed to replicate the combination of kinetic processes from the EIS tests are shown in Figure 14. The solution resistance in this EEC is denoted by Rs, R_Por_ is the porous layer resistance, and R_B_ is the barrier layer resistance. Additionally, for some samples, C_b_ is the capacitance relative to the barrier layer, the constant phase element in relation to the barrier layer is called CPE_B_, and the porous layer constant phase element is called CPE_Por_. The impedance exponents to the porous layer and barrier layer, respectively, are n_Por_ and n_B_, while the Warburg impedance is represented by W_B_. The working electrode is WE.

The roughness and diversity of the porous and passive layers can be represented by the CPE. A phase element’s impedance is described by the parameter ZCPE = [C(i ω))^n^]^−1^, where C is capacitance; i represents current (imaginary number: −1^0.5^); ω is angular frequency; and −1 ≤ n < 1 [73,74]. For 0.5 < n < 1, a distribution of relaxation durations in the frequency space is represented, and a CPE with n = 1 represents an ideal capacitor. It should be noted that CNLS (complex non-linear least squares) simulations were run in order to compare the findings of the simulations with the experimental impedance data. Experimental data were fit using ZView^®^ (version 2.1b) software connected to two EECs, and its quality fitting is confirmed using chi-squared [75,76]. Table 5 and Table 6 show results of the simulations performed using the EEC in Figure 14. The fit data from the EEC model, which closely match the experimental data, are displayed in Figure 12 and Figure 13. According to the EEC simulations, this is the reason why the majority of the samples in Table 5 and Table 6 have error values in the 1.14–4.46 range. Moreover, the low values given by χ^2^ confirm that the suggested EEC models are accurate. Some authors speculate that the reason for this material’s Warburg impedance (W_B_) could be that the high-thickness porous layer becomes brittle, which at first permits ions to pass through quickly. However, once the ions pass through and reach the barrier layer, it becomes difficult for the ions to pass through and accumulate [77]. The Warburg impedance explains the transfer of ions through the porous and passive layers of the coating [78,79]. The Warburg element controls the rate of reaction by allowing oxidizing and reducing species to migrate to and from the metal substrate. In other words, a non-linear diffusion process could originate from the geometric irregularity of the diffusion layers that the coating creates [61]. An increase in WB indicates a change in corrosion kinetics from charge transfer control to diffusion control [80].

The variation in R_s_ is due to the coating morphology, which is related to the surface charge and the electrochemical double layer [81]. They take into account each anodized sample’s pore resistance (R_Por_) at current densities of 3 and 4.5 A/dm^2^ (Table 5 and Table 6). It may be concluded that R_Por_ contributes to the corrosion resistance of anodized materials because it is generally greater in samples than the resistance of the barrier layer (R_B_) provided by the non-anodized material. This leads one to believe that anodizing a material in a citric acid solution rather than conventional anodizing may offer better corrosion protection. The higher CPE_Porr_ and CPE_B_ values for the three non-anodized alloys and the anodized samples at the different solutions and current densities are attributed to thinner porous and barrier layers. The results indicate that the thicknesses of these layers are more relevant than the barrier layer formed naturally by the non-anodized material of the different alloys (see Table 5 and Table 6). Some authors suggest that as the layer’s thickness grows, its capacitance decreases [82,83]. This occurs for the anodized samples of 6061-T6 and 7075-T6 alloys in 1MC 5S and 1MC 10S solutions and the anodizing current densities of 3 and 4.5 A/dm^2^. It is vital to relate the resistance to charge transfer (R_ct_), which is directly proportional to the corrosion resistance and can be related to the R_B_, to the corrosion behavior of anodized materials in order to analyze it. In this way, the material’s corrosion resistance increases with a greater R_B_ value. Analyzing the R_B_ values of the non-anodized and anodized aluminum alloys in citric acid solutions [1M] with different current densities of 3 and 4.5 A/dm^2^ showed the assessment medium’s lowest corrosion resistance, with values ranging from 6.38 × 10^3^ to 531 × 10^3^ Ω-cm^2^; this is mostly because of the extremely thin oxide layer that formed in certain places. In the samples anodized in the 1MS, 1MC 5S, and 1MC 10S solutions, the R_B_ values increased significantly, with values of 362.56 × 10^3^ to 10.20 × 10^7^ Ω-cm^2^, using various densities of anodizing current. According to the EIS results, the various aluminum alloys that were anodized in sulfuric and citric acids have superior corrosion resistance than the non-anodized material in a 3.5 wt.% NaCl solution. The 6061-T6 and 7075-T6 alloy samples in the 1MC 5S and 1MC 10S solutions with the two current densities were the samples that showed greater R_B_ values, presenting values from 6209 × 10^3^ to 1.02 × 10^7^ Ω-cm^2^, which provides excellent corrosion protection.

The impedance brought about by the migration of oxygen and metal ions across the film in samples is referred to as the Warburg element (W). It takes into account the impedance connected to ion transport via the oxide. It is dependent upon the concentration, charge, and diffusion coefficients of the metal and oxygen ions [84]. The level of protection offered by the oxide film increases with the size of the Warburg element (W) [85]. Some authors have drawn a connection between the Warburg impedance and systems’ resistance to diffusion mechanisms penetrating them. In this instance, the resistance of reduced and oxidized species on the metal surface can be understood as the Warburg resistance [86,87,88,89]. Because of this, W_B_ can be interpreted as the anodized coatings’ corrosion resistance in the various citric–sulfuric acid solutions with current densities of 3 and 4.5 A/dm^2^ containing the 6061-T6 and 7075-T6 alloys that exhibited this behavior. The samples that presented diffusive behavior represented by W_B_ were mostly the 6061-T6 and 7075-T6 alloys anodized in 1MC 5S and 1MC 10S solutions, and they presented W_B_ values between 3.00 × 10^6^ and 3.16 × 10^7^ Ω-cm^2^. In these anodizing conditions, the 6061-T6 and 7075-T6 aluminum alloy samples presented the highest W_B_ values; these coatings exhibit the highest level of corrosion resistance among all the samples examined in this study. These findings imply that 2024-T3, 6061-T6, and 7075-T6 aluminum alloys can be hard anodized using citric acid-based solutions, obtaining resistance values that are on par with or greater than those reported in the literature. The anodizing solutions employed in this work efficiently protect aluminum alloys from corrosion and Cl-ion assault, and they are composed of citric acid and minor amounts of sulfuric acid.

## 4. Discussion

The anodizing procedure was performed on various batches of aluminum alloys, including 2024-T3, 6061-T6, and 7075-T6, using baths made of citric acid mixed with trace amounts of sulfuric acid. Table 2 lists the compositions of the various alloys, and Figure 6 and Figure 7 show the chemical composition obtained by SEM-EDS. Space applications, including spacecraft systems, flight hardware, and structures, have made use of these three forms of aluminum and space equipment, including power and onboard systems [90]. The microstructure of the different aluminum alloys affects how the oxide layers grow.

All the anodized samples in the sulfuric and citric acid baths had surface porosity and cracks, and roughly the same morphology was displayed for each solution, regardless of the current density (Figure 2 and Figure 3). In various solutions and current densities of the produced hard anodic coatings, microcracks typical and characteristic of hard-anodized surfaces are detected [91]. The sealing procedure generates thermal stresses that could develop pores and cracks on the material’s surface because the anodic coating and the metallic substrate have different coefficients of thermal expansion. This surface morphology has been seen in alloys AA1050 and AA2024-T3 (aluminum alloys that have been thermally treated, cold worked, and naturally aged) after anodizing with tertiary and boric acid [92,93].

The phase θ (Al_2_Cu) is present in the Al–Cu alloy (2024-T3). The aluminum alloy 6061-T6 contains AlSiFe particles. AlCuMg phases are present in the 7075-T6. According to Figure 4 and Figure 5, the range of oxide thicknesses occurring in the various aluminum alloys is as follows: 7075-T6 < 6061-T6 < 2014-T3. The 2024-T3 aluminum alloy forms the lowest oxide thickness because of its high copper concentration, which lowers current efficiency. Copper oxidation and integration into the anodic film, as well as copper enrichment below the anodic film, are all blamed for the decrease in current efficiency [94,95,96,97,98,99,100]. Because the intermetallic particles Al–Fe–Mn–Si in the 6061-T6 alloy oxidize more slowly than the aluminum matrix, the alloy has a smaller oxide thickness than that generated on the 7075-T6 alloy. They become stuck in the oxide layer, which prevents the oxide from growing [101]. In the 7075-T6 alloy, as it is composed mainly of Al-Zn-Cu-Mg, during the anodizing process, these alloying elements—which are more noble than aluminum—oxidize, facilitating the formation of the oxide film [102,103]. In addition to their impact on oxide formation, intermetallic particles have a significant influence on the anodic layer shape. Depending on their chemical composition, copper-rich phases in the 2024 Al alloy function as either the cathodic or anodic zones. Pitting corrosion is caused by the ϴ phases (Al_2_Cu) acting as cathodic particles and the S phases (Al_2_CuMg) acting as the anodic zone. The surface-formed cavities resemble the morphologies of the intermetallic phases that have undergone dealloying [104]. Because of the fine intermetallic phases that the alloy contains and how they form small voids upon dissolution, alloy 6061-T6 has a homogenous oxide layer (Figure 4 and Figure 5). Due to the presence of coarse Al-Cu and Al-Fe-Cu-Mg-Si intermetallic phases, the 2024-T3 and 7075-T6 alloys have an inhomogeneous oxide layer (Figure 6 and Figure 7). Since Mg, Cu, and Zn are included in the alloys that are most frequently used in the aerospace sector, consideration should be given to how these elements affect the oxide layer that forms during anodizing [105,106]. Si particles and intermetallic compounds rich in Cu, Mg, and Fe are the main barriers inhibiting the formation of an anodic layer in Al alloys. These elements cause the anodized surface to have visually appealing imperfections while also decreasing the oxide layer’s surface mechanical qualities [107]. Intermetallic complexes detrimental to the anodization process include Mg_2_Si, β-Al_5_FeSi, α–Al(Fe, Mn, Cr)Si, and Al_2_Cu phases [108,109]. These secondary phases produce localized changes in the composition and shape of the interface between the bulk material and the oxide layer and have an impact on the thickness of the oxide layer [110]. Moreover, at certain stages, the anodic film’s porosity may rise, while its hardness and thickness may fall [111].

The anodic film’s structure, composition, form, and rate of oxide growth all affect the mechanical characteristics of the anodic layer. The oxide layer generated in the Al 6061-T6 alloy (Figure 9a,b) exhibits the best Vickers microhardness due to small defects created by fine precipitates. However, due to the presence of big defects created by coarse intermetallic phases, alloy 2024-T6 has the lowest Vickers microhardness values in this work, whereas alloy 7075-T6 has intermediate-sized defects and is hence less microhard than alloy 6061-T6. Compared with the oxide layers generated in the 6061-T6 and 7075-T6 alloys, the Al 2024-T6 alloy exhibits a lower microhardness. The strong reactivity of copper-rich intermetallic phases and oxygen production are responsible for the significant flaws and fissures in the oxide layer. Vickers hardness values (VHN, kg/mm^2^) for hard-anodized films are typically found to be between 300 and 500 [112,113]. In this work, only the samples of 6061-T6 alloy anodized with a current density of 3 A/dm^2^ in 1MC 5S and 1MC 10S solution exceeded these values; in contrast, those anodized with a current density of 4.5 A/dm^2^ obtained lower values of microhardness in all alloys. One possible explanation for the microhardness seen in the samples anodized at a current density of 3 A/dm^2^ is the development of boehmite (Al_2_O_3_-H_2_O), which has a hardness ranging from 250 to 600 VH. On the other hand, the microhardness observed in the samples anodized at a current density of 4.5 A/dm^2^ may be linked to the formation of bayerite (Al_2_O_3_-3H_2_O), which is typically microhardened between 150 and 300 VHN [114].

Measurements of cyclic potentiodynamic polarization curves (CPPCs) (Figure 10 and Figure 11) and electrochemical impedance spectroscopy (EIS) are used to characterize the corrosion characteristics of the anodic layers (Figure 12 and Figure 13). The anodized samples’ electrochemical behavior in the various citric acid solutions, where their current densities ranged from 3 to 4.5 A/dm^2^, may be explained by the ways in which hard-ion carboxylates interact with Al^3+^. Complex compounds that are integrated into the surface because of this reaction prevent corrosion [115]. Organic acids’ capacity to build a protective coating by adhering to the metal surface is what gives them their protective effect [116]. In citric acid solution, the H_2_Cit^−^, HCit^2−^, and Cit^3−^ species are potential surface-complexing ligands. The pH of the solution affects the concentration of these ligands (HCit^2−^ species predominated in a pH 6 solution of citric acid, for example). As a result, when an aluminum oxide surface encounters citric acid solutions, a number of distinct surface coordination events take place, producing a variety of surface species that are aluminum-citrate and/or aluminum-hydroxo-citrate. Almost nothing is known about the structure and nature of these surface complexes. Nevertheless, two potential six-membered chelate ring structures of the surface have been observed by several scientists [117,118]. When aluminum is anodized in citric acid and/or citrate solutions, the resulting Al–citrate complexes are formed when the citrate anions are adsorbed on the oxide surface and integrated into the oxide film’s structure. The basic idea of the likely surface coordination events that happen on the surface of aluminum oxide in a citric acid solution of pH range 3–6 is illustrated by Equations (2) and (3), which may be used to explain the potential surface reactions in a pH 6 solution [33,119,120]. Aluminum, Al hydroxide, and different types of Al oxide have extremely little solubility (dissolution) in water and non-complexing solutions [120]. Nevertheless, the minimal solubility is not as well expressed in aqueous complex-forming solutions (such as citrate, oxalate, and salicylate), and surface complexation reactions govern the dissolution [121].

(2) $$\mathrm{Al}-{\mathrm{OH}}_{2}^{+}+{\mathrm{HCit}}^{2-}↔{\mathrm{AlHCit}}^{-}+H_{2}O$$

(3) $$\mathrm{Al}-OH+{\mathrm{HCit}}^{2-}↔{\mathrm{AlHCit}}^{-}+{\mathrm{OH}}^{-}$$

There are two distinct phases in the anodization process of citric acid pore nucleation: Initially, alumina is concurrently deposited and generated at the metal–oxide junction and at the oxide–electrolyte interface to generate a fast-forming barrier-type alumina layer. Current density during the anodizing process produces an electric field that leads to Al^3+^ egress from metal–oxide interfaces and O^2−^/OH^−^ entrance from oxide–electrolyte to metal–oxide surfaces. The electrolyte contains Al–citrate complexes, which stabilize a certain amount of Al^3+^. Second, as the electrolyte Al–citric complex gradually transforms into citric acid-incorporated alumina and deposits it unevenly on barrier-type alumina, an electric field concentration occurs between protuberances and pore growth. It should be noted that the protuberances that arise on the produced alumina surface as a result of the random and delayed integration of alumina by the citric acid cause the electric field to concentrate in particular areas. Because of field-assisted dissolving at the oxide–electrolyte surface, which thins the created flat barrier layer, alumina is produced rapidly at the oxide–metal interface. Pore forming and the preservation of the equifield field strength are caused by the oxide growth at the metal–oxide interface, which is regulated by the morphology at the oxide–electrolyte interface [121].

The thickness of the oxide layer and the presence of various defect types caused by the intermetallic phases have an impact on the anodic layer’s corrosion abilities. The anodic layer of alloy 2024-T3 shows higher corrosion current densities and low barrier layer resistance than alloys 6061-T3 and 7075-T6 due to dissolution of copper intermetallics and oxygen reduction, respectively. The reduced oxide thickness, cracks, and other flaws in the oxide layer are the causes of the anodized Al 2014 alloy’s poor corrosion resistance (Figure 2, Figure 3, Figure 4 and Figure 5). The high corrosion resistance of the anodized 6061-T6 and 7075-T6 alloys under the different conditions of this work is due to the fine size of the precipitates, which minimizes the occurrence of cracks and reduction in the oxide layer thickness (Figure 4 and Figure 5). The pores and cracks present in the anodizing layers of aluminum alloys 2024-T3 and 7075-T6 cause a decrease in corrosion resistance and mechanical properties and, in some cases, thickness of these alloys because Cl- ions are able to enter the base material and degrade it. These pores and cracks are caused by the large intermetallic compounds that are present in these two alloys (see Figure 4a,c,g,i,l and Figure 5a,c,f); in the θ phase (Al_2_Cu) in the case of alloy 2024-T3 and the compounds formed by Al-Cu-Mg for alloy 7075-T6, it can be clearly seen that the intermetallic compounds cause the cracks, and in some of the occasions, these cracks reach the surface of the coating. Alloy 6061-T6 also has precipitates formed mainly by Al-Si-Fe (Figure 4b,e,h,k and Figure 5b,e,h,k); however, these precipitates are small and do not cause cracking or pores during the growth of the anodized layer and therefore have better mechanical properties and corrosion resistance in the anodized samples of this alloy in the different solutions composed of mixtures of citric and sulfuric acid manufactured in this work.

## 5. Conclusions

In aluminum alloys 2024-T3, 6061-T6, and 7075-T6, hard anodizing with sulfuric and citric acid solutions can be applied to produce corrosion resistance and mechanical properties that are level with or better than those of conventional sulfuric acid anodizing. The intermetallic phases found in aluminum alloys affect the anodic layers’ development rate and form. Hard anodizing was achieved with solutions composed mainly of citric acid on aluminum alloys 2024-T6, 6061-T6, and 7075-T6, which in most cases show better mechanical and corrosion resistance properties than the same material conventionally anodized with sulfuric acid. The oxide layers+ generated in the Al 2024-T3 and 7075-T6 alloys, which comprised coarse intermetallic phases, showed large voids and surface flaws. More uniformity and smaller flaws created by fine precipitates were visible in the anodic layer that developed on 6061-T6. The high copper content in the 2024-T3 alloy affects the characteristics of the oxide created during anodizing, while the fine precipitates of Zn, Fe, Mg, and Si present in the alloys 6061-T6 and 7075-T6 do not impede the growth of the oxide film and do not cause defects in the oxide film. The 6061-T6 aluminum alloy was the one that presented the best mechanical properties and corrosion resistance in this work due to the fine size of the precipitates present, followed by the 7075-T6 alloy and finally, the 2024-T3 alloy.

## Figures and Tables

**Table 1:** Parameters of the anodizing process and designations of aluminum alloys.

Alloy	Anodizing				Sealing	Nomenclature
	Current Density (i) (A/dm^2^)	Time and Temperature	Solutions Concentration			
			Citric Acid	Sulfuric Acid		
2024-T3	3	Time: 1 h Temperature: 0 ± 2 °C	[1 M]	---	Deionized water Time: 1 h Temperature: 95 ± 4 °C	3A 1MC
			---	[1 M]		3A 1MS
			[1 M]	5 mL/L		3A 1MC 5S
			[1 M]	10 mL/L		3A 1MC 10S
	4.5		[1 M]	---		4.5A 1MC
			---	[1 M]		4.5A 1MS
			[1 M]	5 mL/L		4.5A 1MC 5S
			[1 M]	10 mL/L		4.5A 1MC 10S
6061-T6	3		[1 M]	---		3A 1MC
			---	[1 M]		3A 1MS
			[1 M]	5 mL/L		3A 1MC 5S
			[1 M]	10 mL/L		3A 1MC 10S
	4.5		[1 M]	---		4.5A 1MC
			---	[1 M]		4.5A 1MS
			[1 M]	5 mL/L		4.5A 1MC 5S
			[1 M]	10 mL/L		4.5A 1MC 10S
7075-T6	3		[1 M]	---		3A 1MC
			---	[1 M]		3A 1MS
			[1 M]	5 mL/L		3A 1MC 5S
			[1 M]	10 mL/L		3A 1MC 10S
	4.5		[1 M]	---		4.5A 1MC
			---	[1 M]		4.5A 1MS
			[1 M]	5 mL/L		4.5A 1MC 5S
			[1 M]	10 mL/L		7 4.5A 1MC 10S

**Table 2:** Aluminum alloys’ chemical composition according to X-ray fluorescence (wt.%).

Alloy	Chemical Elements								
	Al	Cu	Zn	Mg	Si	Fe	Mn	Cr	Ti
2024-T3	Bal.	4.273	0.201	1.012	0.367	0.268	0.494	0.043	0.047
6061-T6	Bal.	0.226	0.195	0.932	0.723	0.454	0.086	0.200	0.338
7075-T6	Bal.	1.641	5.657	2.531	0.224	0.348	0.078	0.166	0.129

**Table 3:** The CPPC electrochemical parameters of various aluminum alloys anodized at 3 A/dm^2^ and submerged in a 3.5 wt.% NaCl solution.

Aluminum Alloy	Current Density and Solution	E_Corr_ (V vs. SCE)	E_A-C_ (V vs. SCE)	E_Pit_ (C vs. SCE)	j_Pass_ (A/cm^2^)	j_Corr_ (A/cm^2^)	Hysteresis
2024-T3	*	−0.656	−0.895	0.256	*	0.34 × 10^−6^	Positive
6061-T6	*	−1.025	−0.858	−0.124	2.60 × 10^−5^	9.47 × 10^−6^	Positive
7075-T6	*	−0.808	−0.945	0.198	*	0.31 × 10^−6^	Positive
2024-T3	3A 1MC	−0.571	−0.900	−0.411	*	1.50 × 10^−6^	Positive
6061-T6		−0.577	−0.762	0.363	*	8.02 × 10^−6^	Positive
7075-T6		−0.688	−0.951	0.283	*	0.11 × 10^−6^	Positive
2024-T3	3A 1MS	−0.664	−0.685	0.404	0.005 × 10^−5^	0.0046 × 10^−6^	Positive
6061-T6		−0.768	−0.792	−0.128	0.0007 × 10^−5^	0.0040 × 10^−6^	Positive
7075-T6		−0.768	−0.768	0.248	0.063 × 10^−5^	0.0296 × 10^−6^	Positive
2024-T3	3A 1MC 5S	−0.453	−0.730	0.511	3.97 × 10^−5^	0.0053 × 10^−6^	Positive
6061-T6		−0.230	0.112	1.006	0.0004 × 10^−5^	0.0003 × 10^−6^	Negative
7075-T6		−0.701	−0.805	0.196	0.861 × 10^−5^	0.085 × 10^−6^	Positive
2024-T3	3A 1MC 10S	−0.526	−0.840	0.407	4.09 × 10^−5^	0.104 × 10^−6^	Positive
6061-T6		−0.551	*	−0.161	0.0002 × 10^−5^	0.0010 × 10^−6^	Positive
7075-T6		−0.739	−0.604	0.307	0.014 × 10^−5^	0.0046 × 10^−6^	Negative

* This value was missing in the respective curve.

**Table 4:** The CPPC electrochemical parameters of various aluminum alloys anodized at 4.5 A/dm^2^ and submerged in a 3.5 wt.% NaCl solution.

Aluminum Alloy	Current Density and Solution	E_Corr_ (V vs. SCE)	E_A-C_ (V vs. SCE)	E_Pit_ (C vs. SCE)	j_Pass_ (A/cm^2^)	j_Corr_ (A/cm^2^)	Hysteresis
2024-T3	*	−0.656	−0.895	0.256	*	0.34 × 10^−6^	Positive
6061-T6	*	−1.025	−0.858	−0.124	2.60 × 10^−5^	9.47 × 10^−6^	Positive
7075-T6	*	−0.808	−0.945	0.198	*	0.310 × 10^−6^	Positive
2024-T3	4.5A 1MC	−0.577	−0.907	0.378	*	48.3 × 10^−6^	Positive
6061-T6		−0.560	−0.792	0.232	*	0.202 × 10^−6^	Positive
7075-T6		−0.716	−0.879	0.177	*	0.51 × 10^−6^	Positive
2024-T3	4.5A 1MS	−0.431	*	0.611	*	0.016 × 10^−6^	Positive
6061-T6		−0.293	0.143	0.978	0.00059 × 10^−5^	0.0012 × 10^−6^	Negative
7075-T6		−0.754	−0.807	0.244	0.89 × 10^−5^	1.15 × 10^−6^	Positive
2024-T3	4.5A 1MC 5S	−0.756	−0.911	0.093	0.012 × 10^−5^	2.51 × 10^−6^	Positive
6061-T6		−0.590	0.065	0.673	0.00040 × 10^−5^	0.00047 × 10^−6^	Negative
7075-T6		−0.679	−0.870	−0.288	0.059 × 10^−5^	0.025 × 10^−6^	Positive
2024-T3	4.5A 1MC 10S	−0.839	−0.971	−0.411	1.57 × 10^−5^	0.297 × 10^−6^	Negative
6061-T6		−0.230	0.150	1.048	0.00053 × 10^−5^	0.0011 × 10^−6^	Negative
7075-T6		−0.811	−0.702	−0.171	1.81 × 10^−5^	0.143 × 10^−6^	Negative

* This value was missing in the respective curve.

**Table 5:** Electrochemical characteristics from Nyquist plots of various aluminum alloys anodized at 3 A/dm^2^ and evaluated in 3.5 wt.% NaCl solution.

Aluminum Alloy	Current Density and Solution	R_Sol_ (Ω·cm)	CPE_Por_ (µF/cm^2^)	n_Por_	R_Por_ (Ω·cm^2^)	CPE_B_ (F/cm^2^)	n_B_	R_B_ (Ω·cm^2^)	W_B_ (Ω·cm^2^/s^0.5^)	Error	χ^2^	EEC
2024-T3	*	28.56	*	*	*	8.63 × 10^−5^	0.73	14.14 × 10^3^	*	<1.42	1 × 10^−2^	1
6061-T6	*	21.61	*	*	*	6.84 × 10^−6^	0.94	0.17 × 10^6^	*	<1.33	3 × 10^−2^	1
7075-T6	*	25.44	*	*	*	2.96 × 10^−5^	0.94	19.84 × 10^3^	*	<1.40	1 × 10^−2^	1
2024-T3	3A 1MC	22.77	1.41 × 10^−6^	0.81	0.559 × 10^3^	4.02 × 10^−5^	0.74	6.38 × 10^3^	*	<1.95	3 × 10^−3^	2
6061-T6		21.57	1.21 × 10^−6^	0.81	1.81 × 10^3^	4.65 × 10^−6^	0.75	18.63 × 10^3^	*	<3.28	1 × 10^−2^	2
7075-T6		30.3	7.29 × 10^−9^	0.77	1.31 × 10^6^	9.80 × 10^−9^	1.00	*	3.16 × 10^7^	<1.42	1 × 10^−2^	3
2024-T3	3A 1MS	24.40	6.68 × 10^−7^	0.77	152.82 × 10^3^	8.41 × 10^−7^	0.94	6209 × 10^3^	*	<1.79	1 × 10^−2^	2
6061-T6		27.28	*	*	*	1.82 × 10^−9^	0.83	3.90 × 10^6^		<1.33	1 × 10^−2^	1
7075-T6		23.5	1.16 × 10^−8^	0.66	1.42 × 10^6^	1.07 × 10^−6^	0.69	2.72 × 10^6^	*	<4.14	6 × 10^−2^	2
2024-T3	3A 1MC 5S	22.77	2.28 × 10^−7^	0.78	46.016 × 10^3^	9.30 × 10^−7^	0.81	5.81 × 10^6^	*	<1.96	1 × 10^−2^	2
6061-T6		14.17	4.67 × 10^−9^	0.77	2.67 × 10^6^	1.04 × 10^−9^	1.00	*	8.63 × 10^6^	<2.35	8 × 10^−3^	3
7075-T6		21.19	2.16 × 10^−7^	0.68	42.58 × 10^3^	1.30 × 10^−6^	0.61	5.12 × 10^5^	*	<2.27	8 × 10^−3^	2
2024-T3	3A 1MC 10S	14.26	8.8 × 10^−7^	0.74	47.54 × 10^3^	3.12 × 10^−6^	0.69	362.56 × 10^3^	*	<1.14	1 × 10^−2^	2
6061-T6		26.34	9.42 × 10^−9^	0.84	2.32 × 10^6^	5.49 × 10^−9^	1.00	*	2.06 × 10^7^	<2.01	3 × 10^−2^	3
7075-T6		16.99	7.54 × 10^−9^	0.75	1.48 × 10^6^	9.80 × 10^−9^	1.00	*	3.16 × 10^7^	<1.42	1 × 10^−2^	3

* This value was missing in the respective curve.

**Table 6:** Electrochemical characteristics from Nyquist plots of various aluminum alloys anodized at 4.5 A/dm^2^ and evaluated in 3.5 wt.% NaCl solution.

Aluminum Alloy	Current Density and Solution	R_Sol_ (Ω·cm)	CPE_Por_ (µF/cm^2^)	n_Por_	R_Por_ (KΩ·cm^2^)	CPE_B_ (µF/cm^2^)	n_B_	R_B_ (KΩ·cm^2^)	W_B_ (KΩ·cm^2^/s^0.5^)	Error	χ^2^	EEC
2024-T3	*	28.56	*	*	*	8.63 ×10^−5^	0.73	14.14 × 10^3^	*	<1.42	1 × 10^−2^	1
6061-T6	*	21.61	*	*	*	6.84 ×10^−6^	0.94	0.17 × 10^6^	*	<1.33	3 × 10^−2^	1
7075-T6	*	25.44	*	*	*	2.96 × 10^−5^	0.94	19.84 × 10^3^	*	<1.40	1 × 10^−2^	1
2024-T3	4.5A 1MC	25.58	5.43 × 10^−5^	0.74	6.55 × 10^3^	4.81 × 10^−4^	0.71	19.47 × 10^3^	*	<1.95	3 × 10^−3^	2
6061-T6		42.86	1.33 × 10^−6^	0.72	4.55 × 10^3^	5.73 × 10^−6^	0.84	27.90 × 10^3^	*	<3.28	1 × 10^−2^	2
7075-T6		43.78	4.32 × 10^−6^	0.57	457.75 × 10^3^	4.60 × 10^−6^	0.64	531.22 × 10^3^	*	<1.42	1 × 10^−2^	2
2024-T3	4.5A 1MS	25.35	6.55 × 10^−7^	0.61	89.04 × 10^3^	1.26 × 10^−6^	0.91	1.02 × 10^7^	*	<1.79	1 × 10^−2^	2
6061-T6		15.20	4.49 × 10^−9^	0.79	1.55 × 10^6^	2.74 × 10^−9^	1.00	*	2.40 × 10^7^	<1.33	1 × 10^−2^	3
7075-T6		38.56	2.09 × 10^−8^	0.63	41.14 × 10^3^	8.68 × 10^−7^	0.95	18.78 × 10^3^	*	<4.14	6 × 10^−2^	2
2024-T3	4.5A 1MC 5S	63.58	6.13 × 10^−7^	0.85	2.67 × 10^3^	6.11 × 10^−6^	0.54	143.83 × 10^3^	*	<1.96	1 × 10^−2^	2
6061-T6		24.73	7.18 × 10^−8^	0.68	301.01 × 10^3^	1.52 × 10^−8^	1.00	*	3.32 × 10^6^	<2.35	8 × 10^−3^	3
7075-T6		51.75	1.61 × 10^−7^	0.61	75.87 × 10^3^	1.11 × 10^−8^	1.00	*	9.23 × 10^6^	<2.27	8 × 10^−3^	3
2024-T3	4.5A 1MC 10S	16.88	1.27 × 10^−6^	0.75	15.65 × 10^3^	1.26 × 10^−6^	0.90	63.23 × 10^3^	*	<1.14	1 × 10^−2^	2
6061-T6		35.38	5.03 × 10^−8^	0.72	409.58 × 10^3^	6.71 × 10^−9^	1.00	*	3.00 × 10^6^	<2.01	3 × 10^−2^	3
7075-T6		17.04	2.07 × 10^−6^	0.68	13.19 × 10^3^	3.12 × 10^−7^	1.00	*	3.74 × 10^6^	<2.70	5 × 10^−3^	3

* This value was missing in the respective curve.

## Data Availability

Data is contained within the article.
